# S-PRG Filler Eluate Induces Oxidative Stress in Oral Microorganism: Suppression of Growth and Pathogenicity, and Possible Clinical Application

**DOI:** 10.3390/antibiotics10070816

**Published:** 2021-07-05

**Authors:** Yu Kono, Muneaki Tamura, Marni E. Cueno, Morio Tonogi, Kenichi Imai

**Affiliations:** 1 Department of Oral and Maxillofacial Surgery I, Nihon University School of Dentistry, Tokyo 101-8310, Japan; deyu18012@g.nihon-u.ac.jp (Y.K.); tonogi.morio@nihon-u.ac.jp (M.T.); 2 Department of Microbiology, Nihon University School of Dentistry, Tokyo 101-8310, Japan; marni.cueno@nihon-u.ac.jp (M.E.C.); imai.kenichi@nihon-u.ac.jp (K.I.)

**Keywords:** oral bacteria, S-PRG filler, oxidative stress, pathogenicity

## Abstract

Controlling the oral microbial flora is putatively thought to prevent not only oral diseases, but also systemic diseases caused by oral diseases. This study establishes the antibacterial effect of the novel bioactive substance “S-PRG filler” on oral bacteria. We examined the state of oxidative stress caused by the six types of ions released in eluate from the S-PRG filler in oral bacterial cells. Moreover, we investigated the effects of these ions on the growth and pathogenicity of Gram-positive and Gram-negative bacteria. We found that the released ions affected SOD amount and hydrogen peroxide in bacterial cells insinuating oxidative stress occurrence. In bacterial culture, growth inhibition was observed depending on the ion concentration in the medium. Additionally, released ions suppressed *Streptococcus mutans* adhesion to hydroxyapatite, *S. oralis* neuraminidase activity, and *Porphyromonas gingivalis* hemagglutination and gingipain activity in a concentration-dependent manner. From these results, it was suggested that the ions released from the S-PRG filler may suppress the growth and pathogenicity of the oral bacterial flora. This bioactive material is potentially useful to prevent the onset of diseases inside and outside of the oral cavity, which in turn may have possible applications for oral care and QOL improvement.

## 1. Introduction

There are many interacting microorganisms in the oral cavity that form the oral microbiota while resisting the host’s immune system [1]. However, once this balance is lost for some reason, it leads to the onset of oral diseases, such as dental caries and periodontal disease [2,3]. In recent years, diseases that develop in the oral cavity are considered to contribute not only to the oral cavity, but also to the development of various systemic diseases [4,5,6], such as diabetes [7] cardiovascular diseases [8], respiratory diseases [9], and Alzheimer’s disease [10]. From this background, control of the oral microbiota (which is also the source of the onset of oral diseases) leads not only to the prevention of oral diseases but also to general health.

The main causes of oral disease development are the formation of bacterial masses attached to the oral tissue, especially the surface of the teeth (called dental plaque or dental biofilm) [11], and the subsequent changes in the bacterial plexus and increased bacterial counts associated to it [12,13,14]. It is most important to suppress plaque formation and maturation in order to prevent onset. Tooth brushing is the most effective way to remove plaque, however it cannot be completely removed by brushing alone. In order to enhance the plaque removal effect, drugs that show various auxiliary effects are used. Nevertheless, as chemotherapeutic agents and disinfectants are often used as the components, there are concerns about the development of resistant bacteria and damage to various tissues in the oral cavity due to long-term use [15,16]. Therefore, there are many reports of new ingredients being used, such as natural antibacterial ingredients, which have been reported as ingredients that cause less damage to the host and can be used for a long time period [17,18,19,20].

Surface pre-reacted glass ionomer (S-PRG) is a new material that releases six types of ions (BO_3_^3−^, Na^+^, Al^3+^, SiO_3_^2−^, Sr^2+^, and F^−^) and is a new bioactive material that can be used repeatedly by charging the ions again, even after the release of ions is completed [21]. The effects of this ingredient are: (1) strengthening tooth structure, (2) suppressing tooth demineralization, (3) buffering capacity against oral acid, (4) suppressing plaque adhesion to resin surface, and (5) beneficial effects on living cells, which have been confirmed [22,23,24]. Currently, S-PRG fillers have already been tested for clinical use by being incorporated into adhesive systems, temporary cements, and orthodontic resins [25,26,27]. Surprisingly, there are few reports that comprehensively examine the antibacterial action against oral bacteria.

In this study, we report on the oxidative stress, stunting, and virulence factor inhibitory effect of the ionic components released from this material on oral bacteria. From these results, the effect of S-PRG as a bioactive substance on oral care was highlighted, and the possibility of preventing not only oral diseases, but also systemic diseases related to oral diseases, was examined.

## 2. Results

### 2.1. SPE Induces Oxidative Stress

To measure SPE filler eluate (ionized water released from S-PRG filler; SPE)-related oxidative stress induction, we measured the amount of superoxide dismutase (SOD) and hydrogen peroxide; both are indicators of intracellular oxidative stress [28]. Levels in *Streptococcus mutans*, *S. oralis*, and *Porphyromonas gingivalis*. *S. mutans* ATCC25175 and *S. oralis* ATCC6249 were used as representatives of Gram-positive and facultative anaerobe bacteria, whereas, *P. gingivalis* ATCC33277 was used as representative of Gram-negative and obligate anaerobe bacterium. These bacteria are indigenous to the oral cavity. *S. mutans* is a lactic acid-producing bacterium that is more involved in the onset of dental caries and *S. oralis* is fast colonizers on oral tissues and a lactic acid-producing bacteria. *P. gingivalis* is the most famous type of periodontal pathogen.

**Figure 1:**
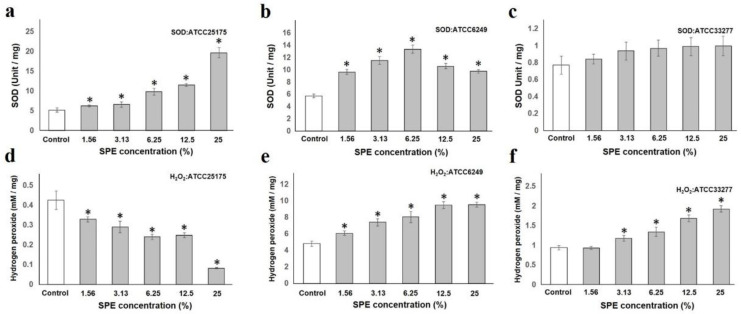
SPE induces oxidative stress. SOD levels in tested bacteria is shown in (**a**–**c**) ((**a**): *S. mutans* ATCC25175, (**b**): *S. oralis* ATCC6249, and (**c**): *P. gingivalis* ATCC33277) compared to test strains with control. Amount of hydrogen peroxide is shown in (**d**–**f**) ((**d**): *S. mutans* ATCC25175, (**e**): *S. oralis* ATCC6249, and (**f**): *P. gingivalis* ATCC33277) compared to test strains with control. Each sample (*n* = 6) indicated the statistical significance of differences between control and treated samples, determined using one-way ANOVA with Scheffe’s test (* *p* < 0.05).

The amount of intracellular SOD increased in both *S. mutans* and *S. oralis*, depending on the concentration of SPE added (Figure 1a,b). There was no significant difference in the SOD level of *P. gingivalis*, however, it increased in a concentration-dependent manner (Figure 1c). In contrast, *S. mutans* hydrogen peroxide levels decreased depending on the concentration of SPE added, and it was considered that the damage of pro-oxidant was small as the amount of hydrogen peroxide decreased. However, the extreme reduction in hydrogen peroxide, which can likewise act as a classical intracellular signaling molecule regulating kinase-driven pathways [29,30,31], has been speculated to possibly cause some metabolic disorder. Both *S. oralis* and *P. gingivalis* hydrogen peroxide level increased according to the added SPE concentration. (Figure 1d–f). Unlike *S. mutan*, *S. oralis* (α-hemolytic bacterial species) has been reported to produce hydrogen peroxide [32,33], and it could be speculated that it is resistant to hydrogen peroxide. In *P. gingivalis*, there was no effect on SOD, only the pro-oxidant effect. This is considered potentially to indicate an increase in intracellular oxidative stress without stimulating the anti-oxidant effect.

These results suggest that the induction of oxidative stress on each test bacterium is different when the SPE dilution concentration is changed.

### 2.2. Bacterial Growth Evaluation

**Figure 2:**
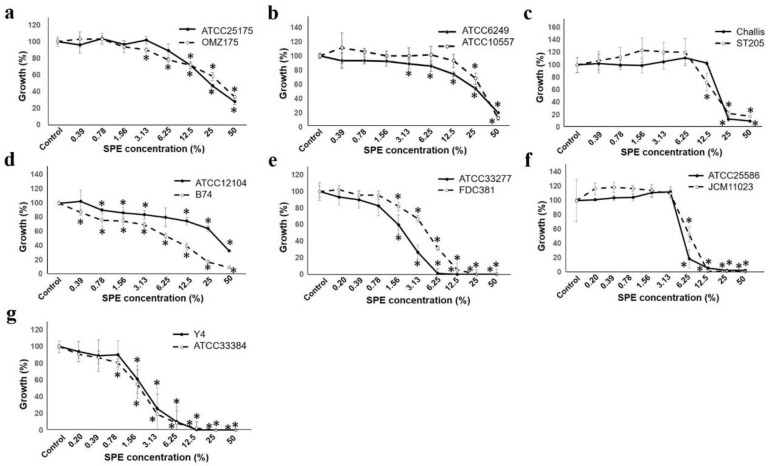
SPE inhibits bacterial growth. Test two strains of each bacteria growth measured at OD600 are indicated. The figure of each alphabet shows the following two strains of bacteria; (**a**): *S. mutans*, (**b**): *S. oralis*, (**c**): *S. gordonii*, (**d**): *A. naeslundii*, (**e**): *P. gingivalis*, (**f**): *F. nucleatum*, and (**g**): *A. actinomycetemcomitans*. The strain number is shown in the upper right of each figure. Result is shown as a ratio to that with 100% control. Each sample (*n* = 6) indicated the statistical significance of difference between control and treated samples, determined using one-way ANOVA with Scheffe’s test (* *p* < 0.05).

To determine SPE effect on bacterial growth, we measured optical density after incubation of the test strain in SPE containing media. Figure 2 shows post incubation turbidity in media containing various SPE concentrations after 24-h incubation. When the growth inhibitory effect on the test bacteria was evaluated by the turbidity method, growth inhibition was observed in all the strains tested in a manner dependent on the added SPE concentration. Figure 2a–d show the Gram-positive

Comparison among the strains (*S. mutans*, *S. oralis*, and *S. gordonii*) showed that each strain had the same tendency on SPE concentration. For *Actinomyces naeslundii*, the graph shows three types on each three strains, and the difference was seen at the highest concentration of 50%.

The Gram-negative bacterial group had a higher antibacterial effect than the Gram-positive bacterial group, and almost no growth was observed at a concentration of 12.5%. *P. gingivalis* was at a 50% concentration, which was almost the same suppression rate, however there was a difference in the tendency up to that point. For *Fusobacterium nucleatum* and *Aggregatibacter actinomyccetemcomitans*, the strains tested showed the same tendency.

Furthermore, experiments conducted by the turbidity method, and the colony forming unit for the antibacterial effect near the SPE concentration of 50% inhibitory effect, and the SPE concentration of MIC50 were calculated and compared (Table 1). Although the concentration was the same, in Gram-positive strains, the colony forming method exerted a growth inhibitory effect at a lower concentration than the turbidity method. In particular, a difference was observed in *S. mutans*; it could be presumed to potentially depend on the medium shape between the liquid and the agar plate [34,35].

### 2.3. Effects on Adhesion and Pathogenic Factors of Test Strains

We investigated not only the growth of oral bacteria, but also the effect on virulence factors on our host.

**Figure 3:**
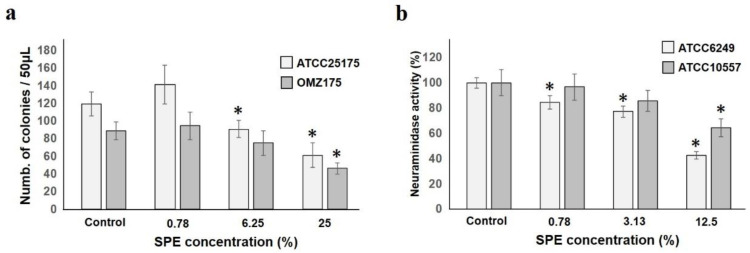
SPE is detrimental to adhesion and neuraminidase activity. (**a**): *S. mutans* ATCC25175 and *S. mutans* OMZ175 adhesion to HA pieces in SPE solution is shown. Colony number with regard to SPE dilution ratio are shown. (**b**): SPE inhibited *S. oralis* ATCC6249 and ATCC10557 neuraminidase activity is shown. Activity is suppressed in a concentration-dependent manner (result is shown as a ratio to that with 100% control). Each sample (*n* = 6) indicated the statistical significance of difference between control and treated samples, determined using one-way ANOVA with Scheffe’s test (* *p* < 0.05).

Adhesion is a phenomenon found in the first stage of the onset of all diseases. Adhesion of *S. mutans* ATCC25175 and OMZ175 to hydroxyapatite (HA) pieces was seen from 6.25%, and was clearly reduced at 25% (Figure 3a). It was speculated that the attachment of these bacteria to the living body was inhibited by SPE.

*S. oralis* ATCC6249 and ATCC10557 neuraminidase activity was suppressed in a concentration-dependent manner and a significant decrease was observed from low concentration, especially in ATCC6249 (Figure 3b). This enzyme is involved in the decomposition of tissue components and the exposure of various bacterial adhesive sites [36]. In this regard, it was confirmed that SPE inhibits enzyme production in addition to suppressing growth.

**Figure 4:**
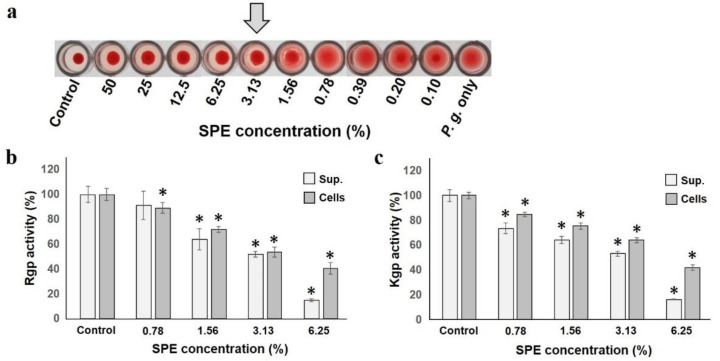
SPE is detrimental to hemagglutination and gingipain activities of *P. gingivalis*. (**a**): Effect of SPE on *P. gingivalis* hemagglutination. The arrow indicates the concentration at which hemagglutination inhibition occurs. (**b**): Rgp gingipain activity in culture supernatant or cells is shown as a ratio to that with 100% control. (**c**): Kgp gingipain activity in culture supernatant or cells. Each sample (*n* = 6) indicated the statistical significance of difference between control and treated samples, determined using one-way ANOVA with Scheffe’s test (* *p* < 0.05).

*P. gingivalis* hemagglutination capacity, which is deeply involved in adhesion and colonization to the host, was inhibited by SPE (Figure 4a). Hemagglutination inhibition started at 6.25%, and was completely inhibited at 3.13%, indicating that SPE suppressed *P. gingivalis* hemagglutination ability. The most famous *P. gingivalis* protease is gingipain, known as Arginine-gingipain (Rgp) and Lysine-gingipain (Kgp) [37]. Both Rgp and Kgp activities produced by *P. gingivalis* were suppressed, depending on the SPE concentration to which both the supernatant and cells were added (Figure 4b,c). In particular, it was confirmed that the addition of a low concentration of 1.56% already significantly suppressed the most important virulence factor of this bacterium.

## 3. Discussion

Oral care is important as both the transition of the oral microbiota and the increase in the number of bacteria are involved in the induction of both oral and systemic diseases [4,5,6]. Tooth brushing is the most important for cleaning the oral cavity, that is, removing plaque. Toothpaste with various antibacterial components has been developed as an aid with certain chemicals. However, some use drugs and disinfectants, and long-term use of such drugs can lead to sudden emergence of drug-resistant strains and damage to the host oral tissue [15,16]. From this study, there are increasing reports of using antibacterial natural plant ingredients and ions instead of harmful ingredients. However, the antibacterial effect of ions on oral microorganisms is not well known, and detailed examination is required. In this regard, this study demonstrates that the ionized water released from the S-PRG filler (SPE) induces oxidative stress on oral bacteria and, likewise, further showed that SPE affects bacterial adhesion activity and pathogenicity.

Oxidative stress has various adverse effects on animals, including human and plant cells, as well as microorganisms [28,38,39,40,41]. Ions are components that give oxidative stress, and it has been reported that each ions (BO_3_^3−^, Al^3+^, SiO_3_^2−^, Sr^2+^, and F^−^) released from the S-PRG filler also causes oxidative stress on animals and plants [42,43,44,45,46,47,48]. We have already reported that the ions released from the S-PRG filler exert oxidative stress on the *Candida albicans*, predominant pathogenic fungi in the oral cavity, and suppress its pathogenicity [49]. In this regard, we hypothesized that SPE treatment might cause oxidative stress in bacterial cells, and measured the amounts of antioxidant SOD and antioxidant hydrogen peroxide in bacterial cells after SPE treatment. As the amount of SOD increased significantly in both *S. mutans* and *S. oralis*, it was considered that active oxygen was produced inside the cells, and there was a difference in the amount of hydrogen peroxide thereafter. This difference is presumed to be due to resistance to hydrogen peroxide [32,33]. However, this difference may require further analysis. The amount of SOD in *P. gingivalis* showed a non-significant increase, however, this bacterium is an obligate anaerobic bacterium, it is considered that the tendency of increasing intracellular SOD amount and the remarkable increase in hydrogen peroxide amount cause considerable damage. Our results of testing typical oral Gram-positive and Gram-negative bacteria suggest that the induction of oxidative stress was thought to be related to the antibacterial mechanism, although there were differences depending on the bacterial species. In this respect, we speculate that this oxidative stress produced by SPE acts to suppress bacterial growth and virulence factors, especially bacterial adhesion and protease production.

SPE exerts oxidative stress on oral bacteria, therefore it could be predicted that SPE is an antibacterial ingredient. The main purpose of antibacterial ingredients is to inhibit the growth of bacteria. Some of the SPE components, especially boron and fluorine, are known to have antibacterial activity [50], which means that SPE can be used directly to inhibit bacterial growth. Throughout the study, we employed seven species and 14 strains of oral bacteria. We found that *S. gordonii*’s character is the same as *S. oralis*, while *A. neaslundii* is related to the accumulation of mature dental plaque, given its high coaggregation activities [51]. *F. nuctearum* is Gram-negative and obligate anaerobe bacterium and related to the accumulation of mature dental plaque same as *A. naeslundii* [52]. *A. actinomycetemcomitans* is a periodontal pathogen and related to aggressive periodontitis. From the experimental results from humidity methods, growth inhibition was observed in all of the tested bacteria in a concentration-dependent manner related to SPE, and there was no difference in the pattern depending on the strain in most of the bacterial species. In particular, SPE could be useful for the control of dental plaque accumulation and protection against dental caries and periodontal disease. Interestingly, the Gram-positive group did not grow at relatively high concentrations, and the Gram-negative group did not grow at concentrations above 12.5%. We postulate that one of the reasons for this is that the obligate anaerobic group is more vulnerable to the oxidative stress produced by SPE than the facultative anaerobic group [28,39]. As some bacteria treated with SPE did not have zero turbidity, MIC50 was measured as one of the antibacterial evaluations. When MIC50 was investigated by the turbidity method and the colony forming method for one strain of each bacterial species, a difference was observed between the two methods and, especially for Gram-positive bacteria, MIC50 concentration by the colony method was lower. This seems to be related to the shape of the medium and the amount of oxygen in the environment. This result suggests that the effect of SPE could differ depending on the growth environment [34,35]. On the other hand, as this material is expected to be added to dental materials and gels as a method of clinical use, it might be considered that the result of solid culture is closer to the clinical effect than the result of liquid culture in consideration of the oral environment.

Bacterial growth is an immediate concern for oral infections, but in the long run, virulence factors should be targeted as well to maintain the health of the host.

*S. mutans* is a bacterial species that is deeply involved in the onset of dental caries [53]. SPE has been reported to suppress the adhesion of *S. mutans* to test tube walls and dentin disc [22,54,55]. In addition, *S. mutans* has been involved since the early stage of dental caries onset, in this case, it must first adhere to the enamel, which is the surface of healthy tooth. *S. mutans* has a glycosyltransferase (GTF) enzyme for producing extracellular polysaccharides, such as dextran and mutan, and there are two types of attachment mechanism of this bacterium: GTF-dependent and GTF-independent for tooth surface. Therefore, when an adhesion experiment was conducted using HA pieces (which are the main components of enamel) the number of adherent bacteria ATCC25175 and OMZ175 was reduced and SPE effectively suppressed *S. mutans* adhesion, which is not dependent on GTF. SPE has been reported to suppress GTF production [56], the main enzyme produced by *S. mutans*, both at the genetic and protein level.

Furthermore, we focused on neuraminidase produced by several streptococcal groups as an enzyme that has a detrimental effect. This enzyme cleaves sialic acid at the end of the surface layer component of oral tissues, exposing the sugar receptor, such as a galactose, that is the attachment site of oral bacteria [36]. When experiments were carried out with two strains of *S. oralis*, which have high neuraminidase activity, activity inhibition was observed in a concentration-dependent manner. This indicates that SPE may reduce the exposure of oral bacteria to glycosyl, such as galactose, acceptors by neuraminidase and suppress bacterial adhesion.

*P. gingivalis* is the most noticeable periodontal pathogen and has various virulence factors. The hemagglutination ability test is an important pathogenic factor for coaggregation of erythrocytes to obtain a nutrient source, especially iron component, without examining the ability of this bacterium to adhere to the biological surface [37]. In this study, sufficient hemagglutination ability was suppressed at a concentration of 3.13%. Furthermore, the effect of this bacterium on the activity of gingipain (a well-known virulence factor) was investigated. Gingipain is a major cysteine protease produced only by *P. gingivalis*, and there are Arg-gingipain (Rgp) and Lys-gingipain (Kgp) with different peptide cleavage site specificities [37]. This enzyme causes the breakdown of biological proteins, damages host cells, and produces various pathological conditions related to periodontal disease. While inducing cell death of gingival fibroblasts and vascular endothelial cells, it is also involved in the survival, proliferation, attachment, and nutrient supply of bacteria. Suppression of hemagglutination and virulence activity proves to suppress the virulence factors of this bacterium and, more importantly, SPE seems to be able to help prevent periodontal disease.

From the above results, it was confirmed that SPE could be useful for suppressing the pathogenicity of oral bacteria regardless of Gram stainability or oxygen requirement.

Additionally, the effect of SPE on suppressing the virulence factors of oral pathogens tested this study may be significant for other phenomena. In particular, it may be very useful to examine the effect of suppressing viral infections such as influenza and COVID-19 in the oral cavity, which is currently a popular topic. Neuraminidase is well known to play a very important role to be released extracellularly after virus replicating inside the host cell in influenza infections [57]. Based on reports that neuraminidase produced by oral bacteria suppresses the effects of neuraminidase inhibitory anti-influenza drugs [58,59], SPE could be predicted to contribute significantly to the treatment of influenza from above results. On the other hand, it is also well known that the pathogenic mechanism (invasion) of protease-dependent viruses is observed when the virus infects the host cell in the first stage [60,61]. At the time of virus infection, after binding to the receptor on the surface of the host cell, the binding site is cleaved with a protease derived from the host or the bacterial flora and the virus invade into the cell. As gingipain of *P. gingivalis* cleaves arginine- or lysine-site of protein, it is speculated that it may be involved in the intracellular invasion of various viruses, including influenza virus. The inhibitory effect of this enzyme may help control the various viruses that infect the above-mentioned infectious systems. There are still many unclear points regarding COVID-19, however, it might be expected that S-PRG filler shows some inhibitory effect. It was speculated that SPE may prevent not only oral diseases, but also systemic diseases, therefore further investigation is needed in the future.

In summary, SPE induced oxidative stress in oral bacteria and showed SPE-related inhibition of several bacterial virulence factors. In this regard, these results indicate that the ions released from the S-PRG filler effectively inhibit both oral bacterial growth and virulence factors. More importantly, it might have potential clinical applications, especially in the prevention of intraoral diseases not only by bacteria, but also by oral viral and extraoral diseases caused by oral microbes. Therefore, S-PRG fillers may be useful in oral care applications, prevention of various oral and oral-related diseases, and improvement of quality of life. In future study, it seems that more detailed examination of other antibacterial mechanism and conditions of use should be conducted assuming clinical application.

## 4. Materials and Methods

### 4.1. Bacterial Cells and Materials

Strains tested this study were *S. mutans* ATCC25175 and OMZ175, *S. oralis* ATCC6249 and ATCC10557, *S. gordonii* Challis and ST202, *A. naeslundii* ATCC12104 and B74, *P. gingivalis* ATCC33277 and FDC381, *F. nucleatum* ATCC25586 and JCM11023, and *A. actinomycetemcomitans* Y4 and ATCC33348.

S-PRG filler, produced according to a previously published method [21] was obtained from SHOFU Inc. (Kyoto, Japan). Briefly, S-PRG filler was mixed with an equal amount of distilled water under constant stirring for 24 h. Subsequently, we centrifuged and used filtered supernatant. Recovered filtrate (S-PRG eluate; SPE) served as the test sample used throughout this study.

### 4.2. Superoxide Dismutase (SOD) and Hydrogen Peroxide Measurement

*S. mutans* ATCC25175, *S. oralis* ATCC6249, and *P. gingivalis* ATCC33277, which are indigenous to the oral cavity, were inoculated with the same broth containing SPE at various dilutions. Briefly, inoculated samples were incubated aerobically or anaerobically at 37 °C for 24 h. Bacterial cells were harvested through centrifugation at 5000× *g* for 10 min under 4 °C chamber conditions and washed twice with Phosphate buffered saline (PBS). Cells (approximately 1.0 × 10^8^) were suspended in 150 μL solution containing 50 mM Tris-HCl buffer (pH 7.6), 1 mM EDTA, and 0.5% triton-X 100. Subsequently, the suspension was placed in ZircoPrep Mini (Nippon Genetics Co. Ltd., Tokyo, Japan) and agitated for 10 min in a Cell disruptor (µT-12, TAITEC, Tokyo, Japan). After sample centrifugation, supernatant was used.

Superoxide Dismutase Assay Kit (Cayman Chemical Company Inc., Ann Arbor, MI, USA) was used to establish SOD amounts in tested bacterial cells, whereas Red Hydrogen Peroxide Assay Kit (Enzo Life Sciences Inc., Farmingdale, NY, USA) was used to establish bacterial hydrogen peroxide amounts following our earlier work [49]. Both kits were performed according to the manufacturer’s recommendation.

### 4.3. Bacterial Growth Evaluation

SPE at the same varying dilutions previously described above was mixed with SG broth. Each mixture was added in a 96-well plate, test strains were inoculated (pre-incubated with Brain heart infusion (BHI; BD, MD, US) broth or GAM (Nissui, Toyo, Japan) broth at 37 °C for 24 h) to a final OD600 = 0.01, and either aerobically or anaerobically incubated at 37 °C for 24 h. Turbidity was measured at OD600 nm using a colorimeter (TriStar LB 941, Berthold Technologies, BadWildbad, Germany) to compare bacterial growth among the test media.

On the other hand, BHI or GAM agar medium, adjusted for each SPE concentration, was prepared and the test bacteria (one strain of each species) were smeared. Afterwards, the medium was cultured under the same conditions as above, the number of colonies formed was counted, and the minimum inhibitory concentration (MIC) of 50 was calculated from the results obtained together with the above turbidity method.

### 4.4. Effects on Adhesion, Hemagglutination, and Pathogenic Factors of Test Strains

To determine the effect on adhesion activity, hydroxyapatite (HA) pieces (3 mm diameter × 2 mm thickness; Cellyard, Pentax, Tokyo, Japan) were used to assess *S. mutans* ATCC25175 and OMZ175 bacterial adhesion. Resin pieces were placed in a 24-well plate while whole saliva was filtered and treated at 56 °C for 1 h and added to each well (1 mL each). Each HA piece was incubated at 37 °C for 1 h to allow saliva film envelopment. Subsequently, HA pieces were washed with PBS, combined with a test bacterial suspension (1.0 × 10^9^ cells/1 mL) containing SPE dissolved at each PBS concentration, and incubated at 37 °C for 1 h. Bacteria non-adhering to the HA pieces were removed by PBS washing and, afterwards, the HA pieces were exposed to ultrasound in 2 mL PBS (20 W, 90 s on ice; Handy Sonic UR-21P, Tomy Seiko Co., Ltd., Tokyo, Japan) in order to release and harvest the adherent bacterial cells. Additionally, suspensions were then diluted, applied to HA agar medium, incubated, and CFU counting was performed [49].

To evaluate the effect on neuraminidase activity, *S. oralis* ATCC6249 and ATCC10557 were employed. After incubating the test bacteria in BHI broth with SPE added at various concentrations at 37 °C for 24 h, supernatant was obtained by centrifugation and filtration. Using the EnzyChrom neuraminidase assay kit (BioAssay Systemes, Hayward, CA, USA), neuraminidase activity was measured by reading 570 nm as recommended by the manufacturer.

To determine the effect on hemagglutination activity, hemagglutination defibrous sheep erythrocytes and *P. gingivalis* ATCC33277 suspended in agglutination buffer were used [62]. Both suspended cells were mixed by changing the SPE concentration, and the agglutination state was visually confirmed after 30 min. To evaluate the effect on gingipain activity, major pathogenic factors of *P. gingivalis* ATCC33277 were tested. The test strain was incubated in GAM broth containing each SPE concentration. Subsequently, we centrifuged and distinguished the supernatant and pellet. Afterwards, supernatant was filtered, and the pellet was crushed as described above. For gingipain activity in supernatant and crushed cell component, Nα-Benzoyl-L-arginine p-nitroanilide hydrochloride (BAPNA; Sigma, Ronkonkoma, NY, USA) was used as a substrate for arginine-gingipain (Rgp) activity [63] and L-Lysine p-nitroanilide dihydrobromide (LyPNA; Sigma, Ronkonkoma, NY, USA) was used for lysine-gingipain (Kgp) activity [64]. After mixing both the sample and substrate, and, likewise, incubating at 37 °C for 20 min, chromaticity was measured at OD405 nm using a colorimeter (TriStar LB 941, Berthold Technologies, BadWildbad, Germany) to compare each degradation activity.

### 4.5. Statistical Analyses

Statistical significance of differences between samples was determined by one-way ANOVA with Scheffe’s test. A significance level of 95% (*p* < 0.05) was considered statistically significant.

## Figures and Tables

**Table 1:** Minimum inhibitory concentration (MIC) 50 SPE concentration in culture medium. MIC50 calculated from the results of the turbidity method and colony forming method of each strain is shown.

	Turbidity Method	Colony Forming Unit Method
Gram Positives		
*S. mutans* ATCC25175	22.9	9.1
*S. oralis* ATCC6249	30.9	20.8
*S. gondorii* Charlis	21.0	14.5
*A. naeslundii* ATCC12104	30.6	25.5
Gram Negatives		
*P. ginigivalis* ATCC33277	2.1	3.1
*F. nuckeatum* ATCC25586	5.0	3.8
*A. actinomycetemcomitans* Y4	2.7	4.5
	Minimum inhibitory concentration 50	SPE concentration (%)

## Data Availability

The data underlying this article will be shared on reasonable request to the corresponding author.
